# Quantifying the Enhancement of Sarcopenic Skeletal Muscle Preservation Through a Hybrid Exercise Program: Randomized Controlled Trial

**DOI:** 10.2196/58175

**Published:** 2024-11-15

**Authors:** Hongzhi Guo, Jianwei Cao, Shichun He, Meiqi Wei, Deyu Meng, Ichen Yu, Ziyi Wang, Xinyi Chang, Guang Yang, Ziheng Wang

**Affiliations:** 1 Graduate School of Human Sciences, Waseda University, Tokorozawa, Japan; 2 AI group, Intelligent Lancet LLC, Sacramento, CA, United States; 3 Chinese Center of Exercise Epidemiology, Northeast Normal University, 5502 Renmin Ave, Nanguan District, Changchun, 130024, China, 81 90-6747-0562; 4 Department of Physical Education, Quanzhou Normal University, Quanzhou, China; 5 Department of Industrial Engineering and Economics, School of Engineering, Tokyo Institute of Technology, Tokyo, Japan

**Keywords:** sarcopenia, older adults, physical exercise program, explainable artificial intelligence, tai chi

## Abstract

**Background:**

Sarcopenia is characterized by the loss of skeletal muscle mass and muscle function with increasing age. The skeletal muscle mass of older people who endure sarcopenia may be improved via the practice of strength training and tai chi. However, it remains unclear if the hybridization of strength exercise training and traditional Chinese exercise will have a better effect.

**Objective:**

We designed a strength training and tai chi exercise hybrid program to improve sarcopenia in older people. Moreover, explainable artificial intelligence was used to predict postintervention sarcopenic status and quantify the feature contribution.

**Methods:**

To assess the influence of sarcopenia in the older people group, 93 participated as experimental participants in a 24-week randomized controlled trial and were randomized into 3 intervention groups, namely the tai chi exercise and strength training hybrid group (TCSG; n=33), the strength training group (STG; n=30), and the control group (n=30). Abdominal computed tomography was used to evaluate the skeletal muscle mass at the third lumbar (L3) vertebra. Analysis of demographic characteristics of participants at baseline used 1-way ANOVA and *χ*^2^ tests, and repeated-measures ANOVA was used to analyze experimental data. In addition, 10 machine-learning classification models were used to calculate if these participants could reverse the degree of sarcopenia after the intervention.

**Results:**

A significant interaction effect was found in skeletal muscle density at the L3 vertebra, skeletal muscle area at the L3 vertebra (L3 SMA), grip strength, muscle fat infiltration, and relative skeletal muscle mass index (all *P* values were <.05). Grip strength, relative skeletal muscle mass index, and L3 SMA were significantly improved after the intervention for participants in the TCSG and STG (all *P* values were <.05). After post hoc tests, we found that participants in the TCSG experienced a better effect on L3 SMA than those in the STG and participants in the control group. The LightGBM classification model had the greatest performance in accuracy (88.4%), recall score (74%), and *F*_1_-score (76.1%).

**Conclusions:**

The skeletal muscle area of older adults with sarcopenia may be improved by a hybrid exercise program composed of strength training and tai chi. In addition, we identified that the LightGBM classification model had the best performance to predict the reversion of sarcopenia.

## Introduction

With the rapid development of medicine and technology, people are increasingly empowered to face life-threatening diseases, which has led to a prolonged human lifespan. However, this has also resulted in a higher prevalence of age-related illnesses. Further, 1 significant issue is the gradual decline in skeletal muscle function, particularly sarcopenia, which is the loss of strength, power, and muscle mass due to aging [1]. Sarcopenia is characterized by decreased muscle strength and impaired regeneration, with individuals potentially losing up to half of their skeletal muscle mass by the age of 80 years [2,3]. This decline is driven by interactions between systemic signaling and intrinsic muscle tissue mechanisms, leading to decreased protein synthesis and myofiber denervation. Additionally, the reduced regenerative potential of aging muscle cannot effectively regulate cell quality [4-6]. These pathological changes in the important part can seriously impact the quality of life and the ability to perform daily activities in older people [7-11]. Sarcopenia is also an objective indicator of cancer cachexia [12] and is associated with suboptimal surgical postoperative outcomes [13], lower survival [12,14], and toxic counteraction [15-17]. Mortality rates nearly double when sarcopenia is combined with inflammation [18]. Therefore, addressing the serious health issues of older people with sarcopenia is imperative.

Many approaches have been proposed to address the muscle loss associated with sarcopenia, including managing chronic low-grade systemic inflammation [19], leveraging the anabolic effects of insulinlike growth factor 1 (IGF-1) signaling [20], and increasing the intake of protein and vitamin D [7]. Correspondingly, hormones, anabolic drugs, and nutritional interventions have been explored for therapeutic applications. It is well established that lean body mass is critical to health, yet the effectiveness of protein interventions in increasing lean body mass remains inconsistent [21,22]. Notably, there is no positive impact on lean body mass or testosterone-induced anabolic responses when protein intake exceeds the recommended intake (0.8 g/kg/day for adults) [23,24]. While treatments such as myostatin antagonists and androgens are being developed and have garnered optimism [25], many commonly prescribed medications have unwanted side effects [25]. Consequently, the Food and Drug Administration has been cautious in approving drugs to treat sarcopenia and has yet to approve any such drug, emphasizing the importance of drug safety. Therefore, the treatment of sarcopenia requires consideration of alternative approaches.

Fortunately, exercise therapy has shown positive effects on older adults with sarcopenia [5]. Specifically, strength exercise training (SET) is regarded as the most effective intervention due to its ability to improve the activation of IGF-1, the Akt/mTOR, and Akt/FOXO3 pathways [26,27]. Additionally, several studies have shown that traditional Chinese exercise also has positive effects on muscle health. Over the last 15 years, 12 traditional Chinese exercise–based studies have been conducted [28-39], with 2 focusing on tai chi [37,38]. Tai chi, which combines physical exercise and respiration, has been shown to decrease fat mass and improve lower limb muscle strength. For example, Yang-style 24-form tai chi over 10 months decreased fat mass [38], while 8-style tai chi significantly improved lower limb muscle strength compared to a control group (CG) [37]. A cross-sectional study on 139 Italian older adults also found that tai chi practitioners had lower body fat content and higher muscle content in the trunk [40]. Altogether, tai chi has demonstrated positive effects on older individuals with sarcopenia. However, it remains unclear whether combining tai chi with SET can further enhance muscle growth and potentially reverse sarcopenia.

In recent years, the increasing interest in applying artificial intelligence (AI) in health care has extended to this field [41,42-44]. Among these applications, a model produced by an explainable AI (XAI) system is particularly noteworthy. This automated diagnostic platform enhances system understandability and trustworthiness through its human-interpreted, high-level learning capabilities. Consequently, this study developed an offline XAI model to forecast whether sarcopenia can be reversed and to boost its clinical applicability.

In summary, this study proposed a novel intervention method for older adults with sarcopenia, combining SET and tai chi. We discuss both the predictive and final effects on participants’ sarcopenia state after 24 weeks of intervention. Based on initial physical ability, computed tomography (CT) scan findings, and 3 types of interventions, the effect was predicted using classic machine learning models. The final effect was quantified and visualized through CT scans. We hypothesized that the combined exercise intervention program of SET and tai chi will more effectively increase muscle mass and reverse sarcopenia than SET alone.

## Methods

### Research Experimental Program

**Figure 1.**
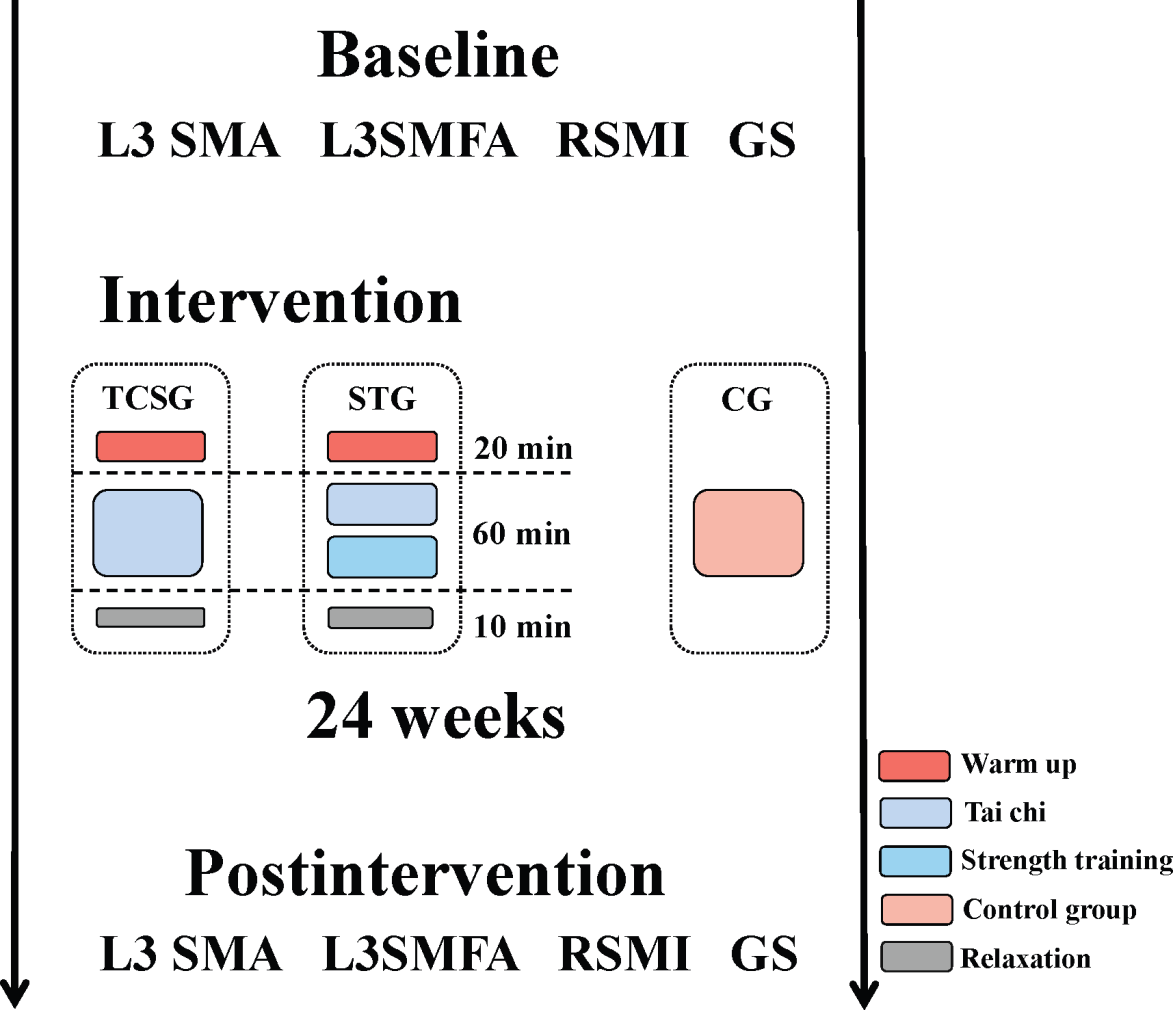
Experimental design flow chart. Participants were assigned to 1 of 3 intervention groups at random: the TCSG, the STG, or the CG. CG: control group; GS: grip strength; RSMI: relative skeletal muscle mass index; SMA: skeletal muscle area; SMFA: skeletal muscle intramuscular fat area; STG: strength training group; TCSG: tai chi exercise and strength training hybrid group.

This research was designed as a randomized controlled and double-blind trial with 3 different intervention groups: a tai chi exercise and strength training hybrid group (TCSG), a strength training group (STG), and a CG. This study was approved by the Ethics Committee of the Northeast Normal University (NC2019030702) and registered at ClinicalTrials.gov (NCT05694117). The random number table method was used to randomly assign participants to the 3 groups. CT provides precise estimates of muscle quality [45], and quantitative CT (QCT) can reveal the level of muscle edema and steatosis. It can also accurately analyze the form and structure of skeletal muscle by removing measurement errors caused by unstable CT readings [46]. Therefore, QCT scans were used to assess the area and density of skeletal muscle at the third lumbar (L3) in each group of participants at the imaging department of Baishan Central Hospital. The training was conducted 3 times a week for 90 minutes, for a total of 24 weeks of intervention from June 1, 2019, to December 30, 2019. The experimental protocol design is shown in Figure 1. During the experiment, each participant was requested to refrain from taking part in any other sort of physical exercise.

### Intervention

In total, 2 experimental groups and 1 CG were set for this trial. The intervention regimens included tai chi exercise hybrid strength training and strength training. The TCSG and the STG were the experimental groups. All participants in the experimental groups started with a 20-minute warm-up exercise and concluded with a 10-minute cool-down exercise. The following is a comprehensive outline of the intervention programs:

1. Tai chi exercise: The TCSG’s tai chi training was structured into 2 cycles. The first cycle (weeks 1‐12) focused on learning and consolidating basic tai chi movements, while the second cycle (weeks 13‐24) aimed at improving and refining these movements. Each tai chi session lasted 30 minutes and included slow, flowing movements combined with deep breathing exercises to promote physical and mental relaxation.
2. Strength training: We designed a total of 5 strength training movements, 2 of which were for training the lower body muscles and 3 for training the upper body muscles. The movements to exercise the upper limb muscles were reverse grip curls, seated pull-downs, and bicep curls. The movements to train the muscle strength of the lower limbs were standing leg raises with an elastic band and supine leg lifts with an elastic band. Further, 1 training cycle lasted for 8 weeks, and the whole training had 3 cycles. In the first cycle, we trained with a light load but many repetitions (from 40% to 60% of 1 repetition maximum [RM] and 12‐20 repetitions). A moderate-intensity load with a medium number of repetitions (60%‐80% of 1 RM and 5‐12 repetitions) was used in the second cycle of training to further enhance the training load. To increase the participants’ maximum muscular strength in the third cycle, we used a greater training load and fewer repetitions (70%‐85% of 1 RM and 5‐8 repetitions). Participants in the STG performed 4 sets of each movement, while the TCSG completed 2 sets with a 2 to 3 minute rest between each set.
3. CG: Participants in the CG were provided with information on sarcopenia treatment and prevention, such as increasing protein intake and engaging in general physical activities.

### Participants

We recruited volunteers from the Baishan Ciming Health Screening Center and the Health Screening Center of Baishan Central Hospital in Baishan, China, for this randomized controlled experiment. A form requesting informed permission was completed by each participant before the beginning of the trial. The screening process involved using the following inclusion and exclusion criteria to choose participants. Inclusion criteria: (1) patients between the ages of 60‐75 years and (2) patients who meet the screening criteria for sarcopenia in Asia established by Asian Working Group for Sarcopenia (AWGS) 2016 [47]. Exclusion criteria: (1) participants taking medications that significantly impact musculoskeletal function, (2) participants enduring respiratory failure or other bodily problems, (3) participants with mental disorders or neurological disorders, and (4) patients who participate in other training programs on a regular basis. Prior research served as the basis for the calculation of the required sample size [48], which was at an α level of .01; we used 80% power and a 0.53 effect, and we assumed a 20% dropout rate. This resulted in a required sample size of 106 participants.

### Assessment of Sarcopenia

The AWGS screening criteria [47] were used for each individual participant’s evaluation. For a participant to be diagnosed with sarcopenia, they must satisfy each criterion listed below:

1. Grip strength: We used a Jamar Hydraulic Hand Dynamometer (SH5001, Saehan Corp, 2017) to measure participants’ grip strength. Participants were instructed to maintain a natural standing posture during the duration of the test. They were also instructed to keep their wrists in a neutral position and their elbows completely extended [49]. The maximum value was retained after they were given 2 separate grip strength tests. The diagnostic boundary values for grip strength that met the screening criteria were <28 kg for males and <18 kg for females.
2. Physical performance: We assessed physical performance using the 6-meter gait speed recommended by AWGS 2016 [47]. Participants prepared behind the starting line, and when they heard the “start” command, they walked at normal speed toward the finish line, taking a few steps after walking across the finish line before stopping to avoid early deceleration [50]. Participants were considered to meet the criteria when their walking speed was less than 0.8 m/s.
3. Appendicular skeletal muscle mass (ASM): We used a multifrequency bioelectrical impedance analysis (Inbody S10 Biospace, Biospace Co Ltd) to measure the ASM. The screening criteria were satisfied by the men and women whose ASM was less than 7 kg/m^2^ and less than 5.7 kg/m^2^, respectively.

### Assessment Methods for Skeletal Muscle and Fat

#### Overview

Further, 1 week prior to the beginning of the intervention and 1 week after it had concluded, we used a GE Revolution 256-row CT (General Electric Company, 2015) to measure the participants’ abdomens. The scan was performed from the top of the diaphragm to the level of the umbilicus, and it was performed with the patient in a supine position with the hands raised flat over the head and the breath held at the end of a deep inspiration. The Gemstone Spectral Imaging scan used tube voltages ranging from 80 to 140 kVp (Peak kilovoltage), currents in the tubes that were intelligently regulated, a layer thickness of 5 millimeters, and a layer spacing of 5 millimeters. Immediately after the scanning process, the data and photos were saved in an automated manner in the workstation. The region of interest was manually defined at the L3 level using the X Section software on the GE ADW (version 4.7) workstation.

#### Measurement of Skeletal Muscle

Skeletal muscle density at the L3 vertebra (L3 SMD; Hounsfield units [HU]) and skeletal muscle area at the L3 vertebra (L3 SMA; cm^2^) were measured using abdominal CT, with the HU range from −30 to 150 HU. This range provides the most realistic measurement of muscle and fat mass [51]. After a scan, an X Section software was used to manually outline the edges of the skeletal muscle tissue at this level, and the software automatically calculated the area of the corresponding tissue within the outlined area and the mean value of the CT.

#### Measurement Method for Skeletal Muscle Intramuscular Fat

Skeletal muscle intramuscular fat accumulates as the muscle mass decreases during the aging process [52]. At baseline and after the intervention, QCT was used to quantify the skeletal muscle intramuscular fat density at the L3 vertebra (L3 SMFD; HU), and the skeletal muscle intramuscular fat area at the L3 vertebra (L3 SMFA; cm^2^). We used a range from −200 to 0 HU for the quantitatively measuring the fat threshold. After scanning, the X Section software was used again to manually outline the edges of the skeletal intermuscular fat at this level, and the software automatically calculated the area of corresponding tissue within the outlined area and the mean value of the CT.

#### Measurement Method for Relative Skeletal Muscle Mass Index

A relative skeletal muscle mass index (RSMI; kg/m^2^) could be used to assess muscle growth [53]. The RSMI, which is represented as muscle mass per m^2^ of the limbs, can be used to evaluate sarcopenia. Thus, RSMI was chosen as the main result indicator in the research and was evaluated using multifrequency bioelectrical impedance analysis.

#### Measurement Method for Muscle Fat Infiltration

According to research, muscle fat infiltration (MFI; %) will cause a reduction in muscle mass, producing muscular atrophy and leading to sarcopenia [54]. Therefore, we used the MFI as one of the observed indicators of the results as well. MFI was calculated by Equation 1:


(1) MFI=SMFA/(SMA+SMFA)\times 100

#### Measurement Method for Grip Strength

Grip strength is the most important criterion for diagnosing sarcopenia and a common indirect measure of total muscle strength [55]. Therefore, grip strength was selected as the primary observation and measured before and after the intervention. We used a Jamar Hydraulic Hand Dynamometer to measure grip strength of participants. Participants were instructed to maintain a natural standing posture during the duration of the test. They were also instructed to keep their wrists in a neutral position and their elbows completely extended [49]. The maximum value was retained after they were given 2 separate grip strength tests.

### Data Analysis

In order to make the intervention runs as accurate as possible, we used a machine learning method to predict whether older people had sarcopenia after 24 weeks of intervention based on the participants’ initial status before the intervention and the intervention protocol they received as features. The data used to train the model consisted of each participant’s age, gender, and initial grip strength, RSMI, L3 SMA, and L3 SFMA as features and the presence or absence of sarcopenia after the intervention as a label. The performance of classification models was assessed per the *F*_1_-score, precision, area under the curve (AUC), recall, and accuracy. These metrics were averaged across all validation or test sets during the repeated cross-validation process, with SD used to provide uncertainty estimates for these averages. Each model was trained 100 times individually using a 10-fold cross-validation. Hyperparameter tuning was performed using a grid search with cross-validation within each training fold to identify the optimal parameters for each model. For example, for the random forest (RF) model, we varied the number of trees (50 to 200), maximum depth (5 to 20), and minimum samples split (2 to 10). The best model was defined as the one with the highest average AUC across all folds. We also calculated the precision, recall, and *F*_1_-score using a threshold of 0.5 for class predictions, ensuring consistency in performance evaluation. First, we trained 9 machine learning classification models using a k neighbors classifier [56], logistic regression (LR) [57], a gradient boosting classifier [58], linear discriminant analysis [59], an extra tree classifier [60], an RF classifier [61], a decision tree classifier [62], an XGBoost classifier [63], and a LightGBM classifier [64]. Then, the 3 best-performing models were selected for the stacking model in this dataset. The first layer consisted of the LightGBM, XGBoost, and RF classifiers. The second-level classifier, used to combine the outputs of these 3 models, was LR. In this research, a stacking technique was used in order to integrate numerous classifiers that were produced by various algorithms *L_1_,...,L_n_* and applied to a singular dataset S. This dataset included instances of the type *S_i_* = (*x_i_, y_i_*), where *x*_*i*_ indicates the characteristic vectors and *y_i_* indicates the classifications. In the beginning of this procedure, a group of base-level classifiers*—C_1_*, *C_2_*, and *C_3_*, together with Ci = *L_n_*(*S*)—were developed. Then, we had predictions for *S_i_*, as per Equation 2:


(2) $${\hat{y}}_{i}^{k}=C_{k}^{i}(x_{i})$$

The meta-level data were made up of several illustrations of the form ((${\hat{y}}_{i}^{1}$,..., ${\hat{y}}_{i}^{n}$), $y_{i}$), in which features represent the output of the first-level classifier and categories are the appropriate labels for this sample.

**Figure 2.**
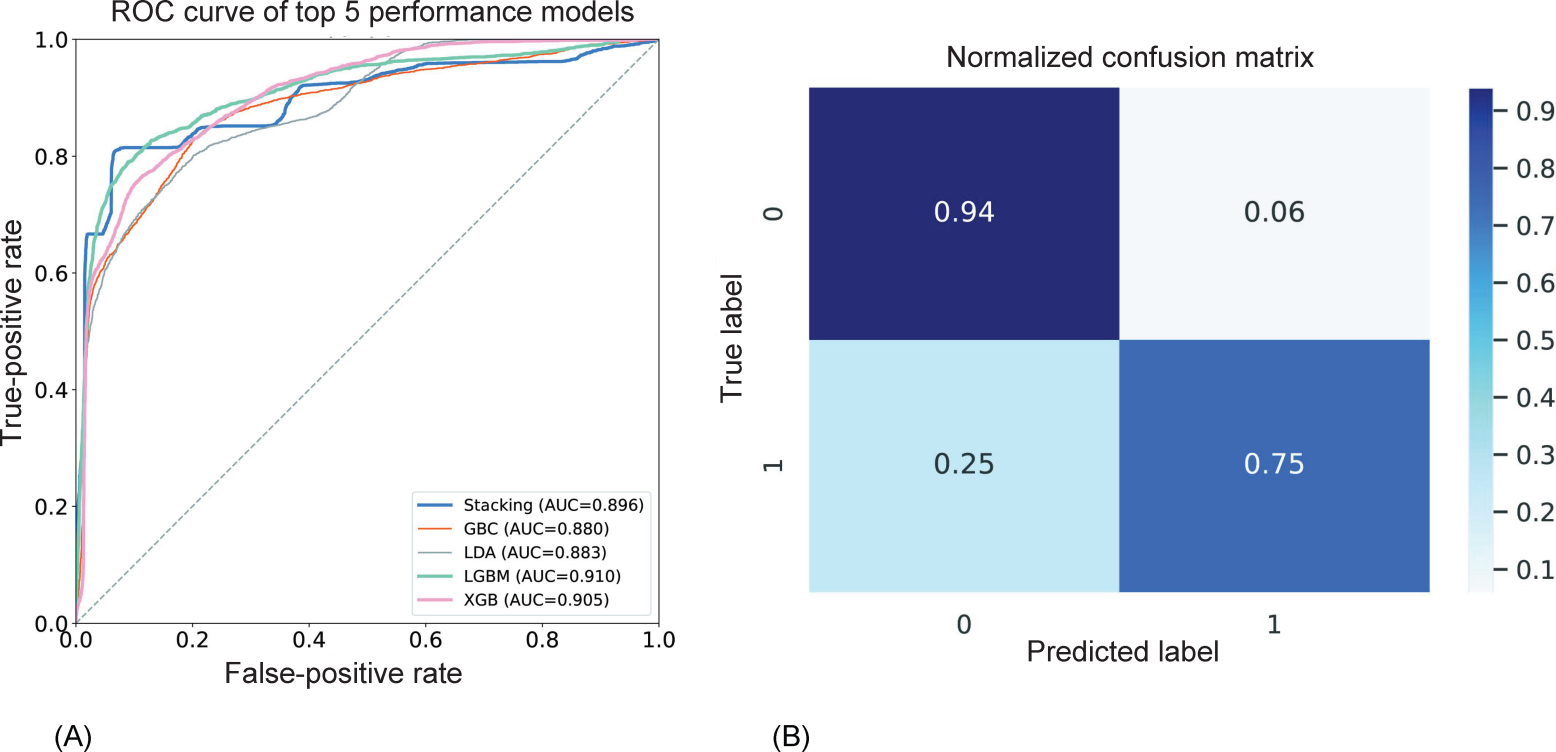
The results of the model’s performance to predict whether a participant is a patient with sarcopenia at second evaluation; the ROC curve of the top 5 models, and the confusion matrix of the LightGBM classifier model. (A) The top-5 performance models’ ROC curve; (B) normalized confusion matrix. AUC: area under the curve; GBC: gradient boosting classifier; LDA: linear discriminant analysis; LGBM: LightGBM classifier; ROC: receiver operating characteristic; XGB: XGBoost classifier.

The dataset was divided into 10 folds for cross-validation. Each model was trained on 9 folds and validated on the remaining fold, repeated 10 times for a total of 100 repetitions. For each fold, the model was trained and predictions were made on the validation fold. These predictions were saved. For each cross-validation run, the precision, recall, accuracy, and *F*_1_-score were calculated. The means and SDs of these metrics over the 100 repetitions are reported in Table 1, providing an estimate of model performance and its variability. Predictions from all 100 cross-validation runs were aggregated to form a single prediction set. This aggregated prediction set was then used to generate the normalized confusion matrix presented in Figure 2, ensuring a comprehensive evaluation of the model’s performance.

**Table 1.** Comparison of the performance of machine learning classification models. Performance metrics are reported as mean (SD), calculated over 100 repeats of 10-fold cross-validation.

Models	Accuracy (%), mean (SD)	Precision (%), mean (SD)	Recall (%), mean (SD)	*F*_1_-score (%), mean (SD)
KNN^a^	78.6 (1.9)	65.7 (5.9)	61.9 (5.2)	61.6 (4.2)
Decision tree	80.6 (2.5)	69.4 (6.7)	70.2 (5.7)	66.4 (5)
Logistic regression	82.8 (1.4)	75.9 (6.8)	60.6 (3.9)	63.6 (3.3)
Gradient boosting	83.5 (1.9)	76.5 (5.9)	67.1 (4.1)	67.9 (4.2)
LDA^b^	84.4 (1.6)	78 (4.9)	68 (3.4)	69.3 (3.7)
Extra tree	84.9 (1.8)	80.6 (7.1)	65.6 (4.8)	68.4 (4.9)
Random forest	85.5 (1.8)	81.7 (7.9)	60.3 (4.3)	65.7 (5.2)
XGBoost	85.8 (1.7)	81.4 (5.5)	68 (4)	71.2 (3.5)
Stacking	86.6 (2.6)	83.9 (5)^c^	67 (1.8)	71.3 (2.3)
LightGBM^c^	88.4 (2)^c^	83.9 (6.1)	74 (4.5)^c^	76.1 (5.1)^c^

a KNN: k neighbors classifier.

b LDA: linear discriminant analysis.

c The values indicate the best performance achieved by each model for the corresponding metric.

Moreover, the receiver operating characteristic curves for the top 5 models are shown in . The horizontal axis depicts false-positive rate, while the vertical axis depicts true-positive rate. A greater AUC indicates superior performance. The LightGBM classifier model had the highest AUC (0.910). The normalized confusion matrix offers a more perceptible view of the accuracy of each category’s predictions made by the model, while the prediction result confusion matrix clearly displays all model predictions, with the real labels in the horizontal direction and the outcomes predicted by the model in the vertical direction. It is clear from that the model showed great performance in predicting whether or not participants reversed sarcopenia after the intervention (94% and 75%, respectively).

In addition, we calculated SHAP (Shapley additive explanations) values, because it could assign an important value to each feature that represents the effect on the model prediction of including that feature [65]. Any machine learning model’s output can be interpreted using SHAP, a game-theoretic approach. The SHAP value quantifies the contribution of each feature to the model’s predictions, represented by Equation 3:


(3) $$\phi_{j}=\sum_{S_{F}\subseteq F\setminus \left\{j\right\}}\frac{\left|S_{F}\right|!\left(\left|F\right|-\left|S_{F}\right|-1\right)!}{\left|F\right|!}\left[f_{S_{F}\cup \left\{j\right\}}\left(x_{S_{F}\cup \left\{j\right\}}\right)-f_{S_{F}}\left(x_{S_{F}}\right)\right]$$

in which *S_F_* stands for all of the potential subsets that do not contain characteristic *j, x* stands for the values of the input characteristics, |*S_F_*| stands for the dimension of *S_F_*, and *j* stands for a specific characteristic. To calculate this effect, 2 models, $f_{S_{F}\cup \left\{j\right\}}$ and $f_{S_{F}}$, were trained, conditioned on the presence or absence of feature j throughout the training process. This allows the contribution of each feature to be calculated. We used the SHAP TreeExplainer algorithm to identify important features that could accurately predict whether participants could reverse muscle loss after the intervention.

### Statistical Analysis

All data analysis, statistical analysis, and visualization procedures for this study were completed using Python (version 3.7.1; Python Software Foundation) and SPSS (version 25.0; IBM Corp) software. Before performing ANOVA, data distribution was assessed using the Shapiro-Wilk test to check for normality, and Levene test was used to check for homoscedasticity. Nonnormally distributed data were log-transformed. Further, 1-way ANOVA and *χ*^2^ tests were responsible for assessing baseline differences in demographic data among groups. Repeated-measures ANOVA was used to examine the impact of the 3 different intervention programs on the participants. Simple effects were used for the factors that had further interaction effects. The Bonferroni post hoc test was used to further investigate group differences when ANOVA indicated statistical significance. The Bonferroni post hoc test was chosen for its control of the family-wise error rate, making it suitable for pairwise comparisons. A *P* value of <.05 was used as the threshold for statistical significance

### Ethical Considerations

This study was conducted in accordance with the Declaration of Helsinki and approved by the Ethics Committee of Northeast Normal University (approval NC2019030702). A written informed consent form was obtained from each participant. Measures were implemented to safeguard participants’ privacy during data analysis.

## Results

### Overview

**Figure 3.**
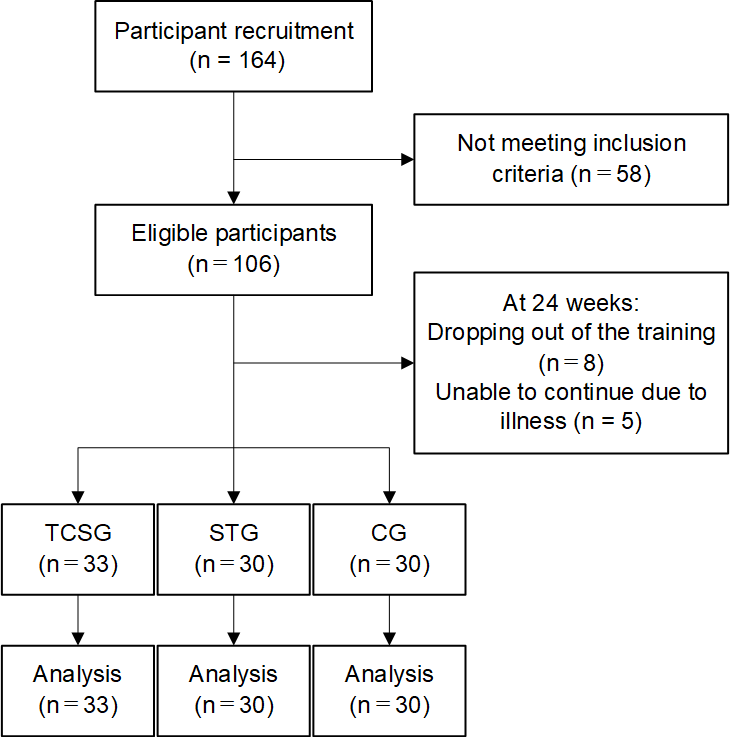
CONSORT flow diagram. CG: control group; CONSORT: Consolidated Standards of Reporting Trials; STG: strength training group; TCSG: tai chi exercise and strength training hybrid group.

A total of 164 individuals were recruited (Figure 3); however, only 106 (48 females and 58 males) were found to meet the inclusion and exclusion criteria. Finally, 93 (50 females and 43 males) participants finished the research. Dropping out of the training (n=8) and being unable to continue due to illness (n=5) were the reasons for not finishing the program. The demographic characteristics of the participants at baseline are summarized in Table 2.

**Table 2.** Demographic features of the participants at baseline.

Items	TCSG^a^ (n=33)	STG^b^ (n=30)	CG^c^ (n=30)	*P* value
Gender, n	male: 14; female: 19	male: 13; female: 17	male: 16; female: 14	.64
Age (years), mean (SD)	66.94 (4.42)	66.87 (3.84)	65.42 (3.97)	.34
BMI (kg/m^2^), mean (SD)	23.23 (2.06)	22.8 (3.18)	21.93 (2.86)	.79

a TCSG: tai chi exercise and strength training hybrid group.

b STG: strength training group.

c CG: control group.

**Figure 4.**
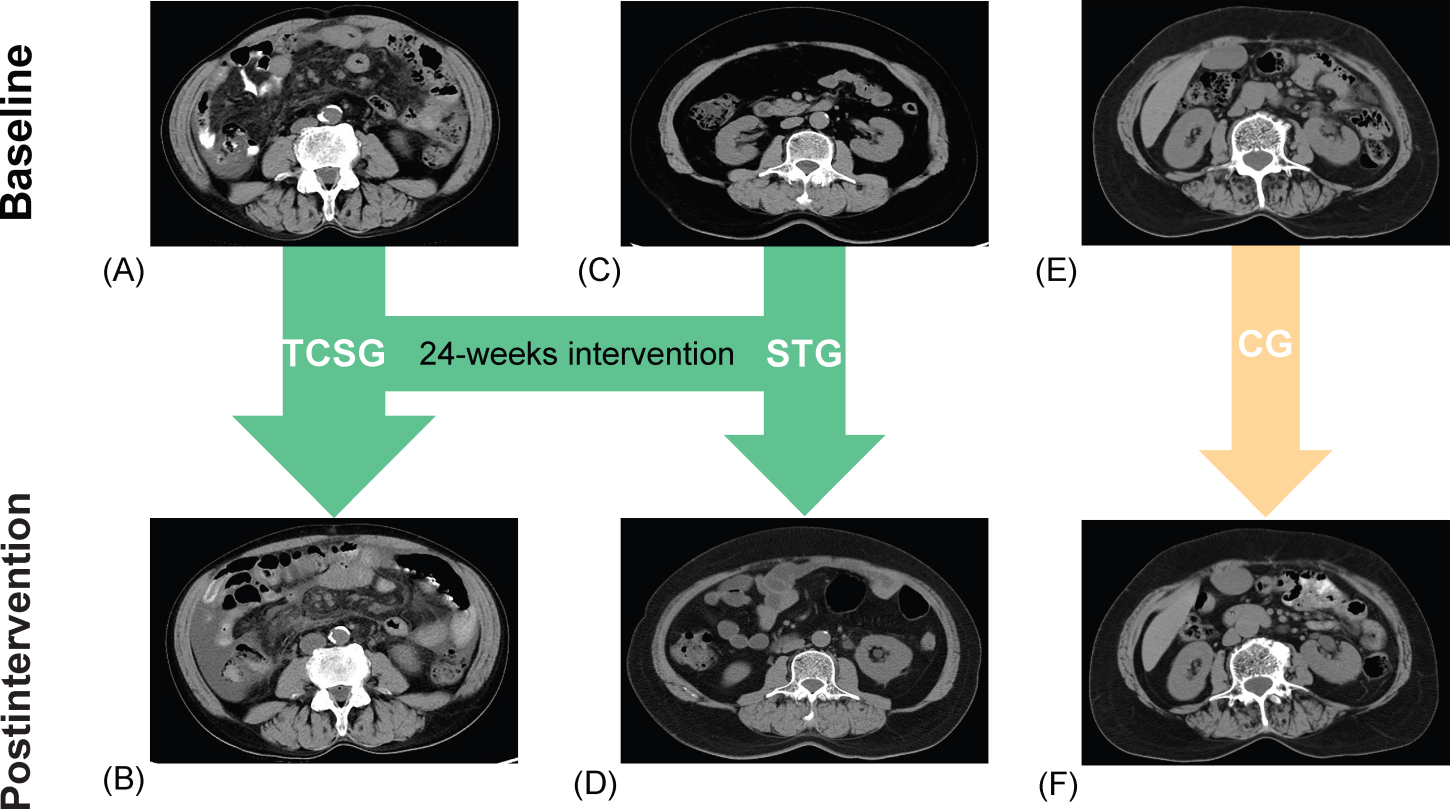
Shows the results of QCT scans of representative participants in each group before and after receiving the intervention. (A) Preintervention TCSG, (B) postintervention TCSG, (C) preintervention STG, (D) postintervention STG, (E) preintervention CG, and (F) postintervention CG. CG: control group; QCT: quantitative computed tomography; STG: strength training group; TCSG: tai chi exercise and strength training hybrid group.

Further, a week after the experiment finished, we reassessed participants for sarcopenia. In the TCSG, we observed the reversal of sarcopenia in 15 (45.5%) participants, and in the STG, we observed the reversal of sarcopenia in 12 (40%) participants, for a total of 27 (29%) with reversal of sarcopenia. Expectedly, sarcopenia was not reversed in the CG participants. Furthermore, Figure 4 shows the QCT measurements of representative participants in each group before and after the intervention.

### Statistical Analysis Results

**Figure 5.**
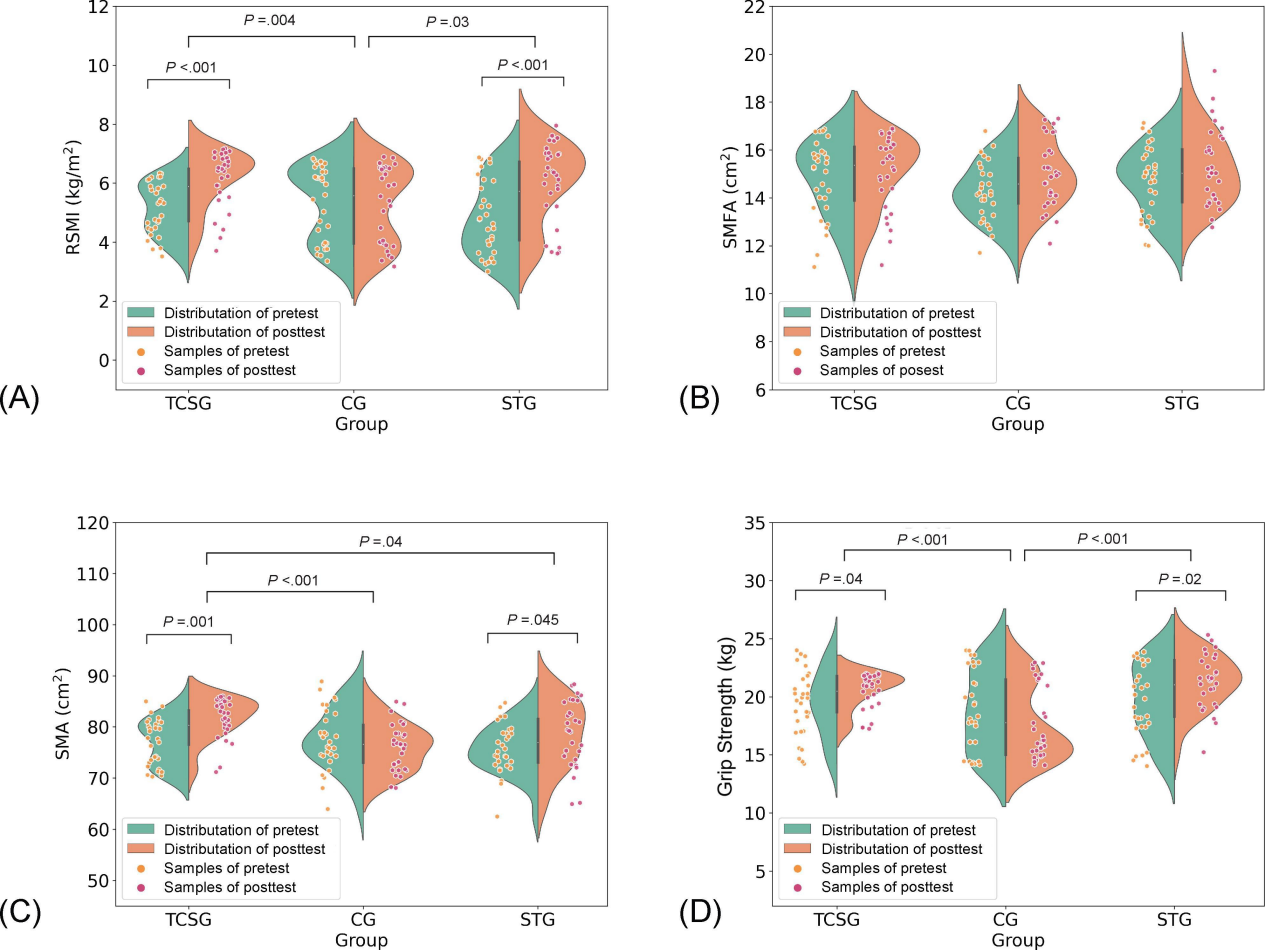
Violin plots of main results before and after the intervention for each group of participants. (A) RSMI; (B) L3 SMFA, (C) L3 SMA; and(D) grip strength. Violin plots show the distribution of the data, with the width of the plot indicating the density of data points at each value. Thex-axis represents the intervention groups (TCSG, STG, and CG), and the y-axis represents the measured outcomes (RSMI, L3 SMFA, L3 SMA, gripstrength). The green area represents the distribution of pretest values, and the orange area represents the distribution of posttest values. Individualpretest samples are marked by orange dots, and posttest samples are marked by red dots. CG: control group; RSMI: relative skeletal muscle massindex; SMA: skeletal muscle area; SMFA: skeletal muscle intramuscular fat area; STG: strength training group; TCSG: tai chi exercise and strengthtraining hybrid group.

Figure 5 depicts the main results, and Table 3 presents results of repeated-measures ANOVA for each observation indicator. All variables did not differ between groups at baseline. It was found that grip strength (*P*=.008), RSMI (*P*=.002), L3 SMD (*P*<.001), L3 SMA (*P*=.005), and MFI (*P*=.008) had a statistically significant interaction effect. A significant improvement in grip strength, RSMI, L3 SMA, and L3 SMD was seen in the TCSG and STG at 24 weeks. There was a significant difference in postintervention L3 SMA in the TCSG compared to the STG and CG, as determined by a post hoc test. However, we did not find any significant interactions among L3 SMFA and L3 SMFD.

**Table 3.** Comparison of intervention effects between groups. Repeated-measures ANOVA analysis.

Parameters	TCSG^a^ (n=33)		STG^b^ (n=30)		CG^c^ (n=30)		Group × time^d^
	Baseline	24 wk	Baseline	24 wk	Baseline	24 wk	*P* value
RSMI^e^ (kg/m^2^)	5.10 (0.91)	6.22 (0.93)^f^^,^^g^	4.84 (1.23)	6.05 (1.30)^f^^,^^g^	5.32 (1.21)	5.24 (1.28)^f^	.002
SMFA^h^ (cm^2^)	14.91 (1.59)	15.15 (1.49)	14.73 (1.43)	15.30 (1.58)	14.28 (1.24)	15.03 (1.39)	.38
SMA^i^ (cm^2^)	77.01 (4.42)	81.83 (3.83)^f^^,^^j^	75.79 (4.77)	78.64 (6.36)^f^^,^^k^	77.63 (5.81)	77.06 (4.53)^f^	.005
GS^l^ (kg)	19.28 (2.75)	20.60 (1.50)^f^^,^^k^	19.73 (3.13)	21.35 (2.29)^f^^,^^k^	18.72 (3.48)	17.67 (3.10)^f^	.008
SMD^m^ (Hounsfield units)	32.30 (1.84)	34.60 (1.98)^f^^,^^g^	32.64 (3.03)	34.72 (2.80)^f^^,^^g^	32.69 (3.72)	32.44 (3.31)^f^	<.001
SMFD^n^ (Hounsfield units)	−65.14 (4.23)	−65.35 (3.73)	−64.01 (5.43)	−64.48 (4.71)	−64.21 (5.69)	−64.68 (6.11)	.76
MFI^o^ (%)	16.23 (1.75)	15.61 (1.30)	16.30 (1.61)	16.35 (1.93)	15.60 (1.82)	16.52 (1.52)^k^	.008

a TCSG: tai chi exercise and strength training hybrid group.

b STG: strength training group.

c CG: control group.

d Analysis of two-way repeated measures ANOVA

e RSMI: relative skeletal muscle mass index.

f Significant difference among groups (*P*<.05).

g Significant difference within the group before and after intervention (*P*<.001).

h SMFA: skeletal muscle intramuscular fat area.

i SMA: skeletal muscle area.

j Significant difference within the group before and after intervention (*P*<.01).

k Significant difference within the group before and after intervention (*P*<.05).

l GS: grip strength.

m SMD: skeletal muscle density.

n SMFD: skeletal muscle intramuscular fat density.

o MFI: muscle fat infiltration.

### Results of Machine Learning Model Classification

The average performance evaluation of the machine learning model after 100 rounds of training is shown in Table 1. We found that the LightGBM classification model exhibited the best performance in terms of average accuracy (88.4%, SD 2%), average recall (74%, SD 4.5%), and average *F*_1_-score (76.1%, SD 5.3%). In addition, our stacking model with the first layer consisting of LightGBM classification model, XGBoost classification model, and RF classification model, and the second layer consisting of LR exhibited the best average precision (83.9%, SD 5%).

Moreover, the receiver operating characteristic curves for the top 5 models are shown in Figure 2A. The horizontal axis depicts false-positive rate, while the vertical axis depicts true-positive rate. A greater AUC indicates superior performance. The LightGBM classifier model had the highest AUC (0.910). The normalized confusion matrix offers a more perceptible view of the accuracy of each category’s predictions made by the model, while the prediction result confusion matrix clearly displays all model predictions. This was with the real labels in the horizontal direction and the outcomes predicted by the model in the vertical direction. It is clear from Figure 2B that the model showed great performance in predicting whether or not participants reversed sarcopenia after the intervention.

### Results of Feature Contribution

**Figure 6.**
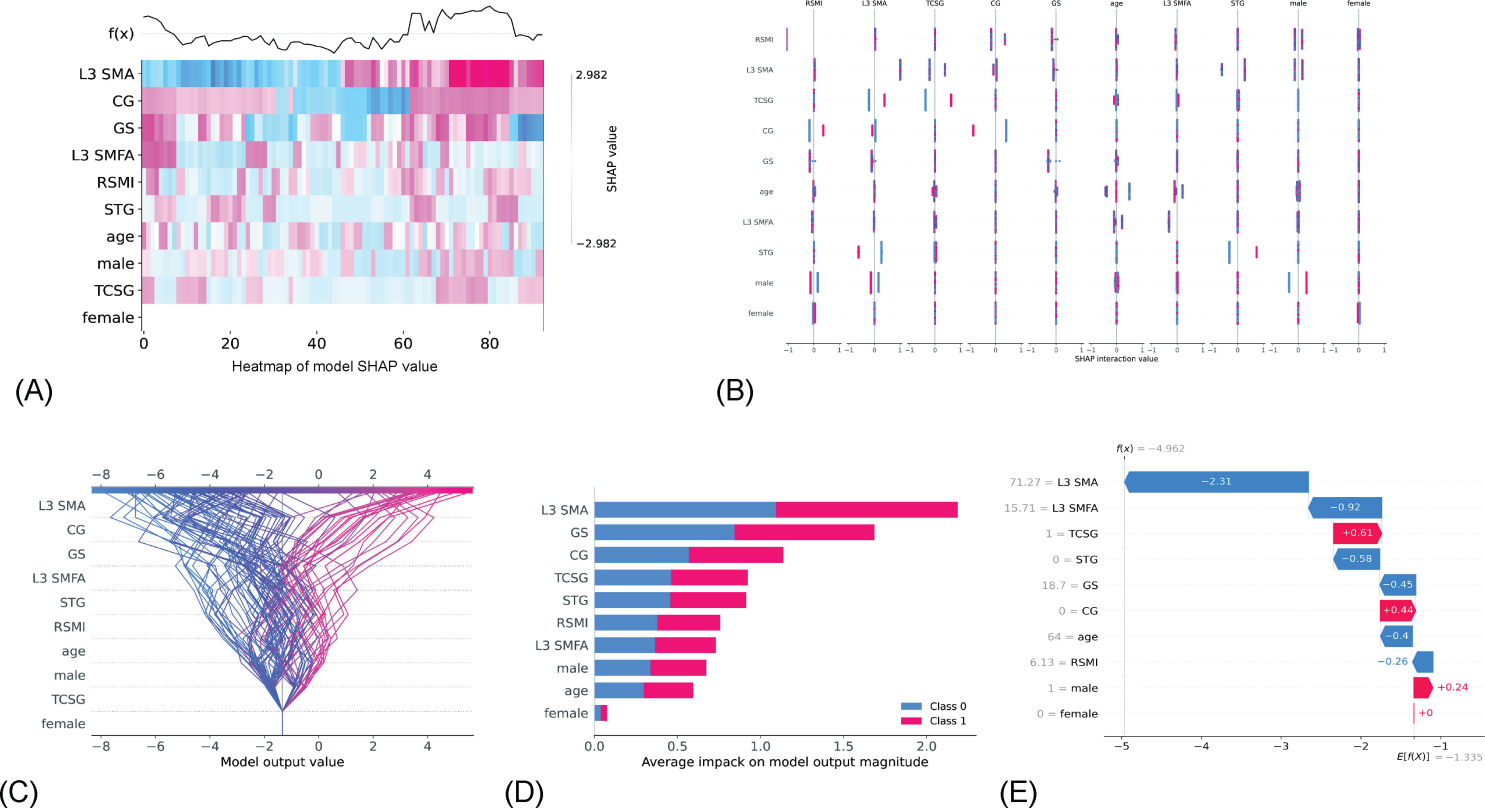
The SHAP values for each feature are shown in this plot. The various characteristics are shown along the y-axis. (A) Heat map plot: shows the SHAP values for each feature; the x-axis denotes the sample sequence; red denotes positive impacts and blue represents negative impacts; the degree of contribution is determined by the color shade. *f(x*) denotes the output of before activation function. (B) Interaction values plot: illustrates the connection that exists between the various features. (C) Decision plot: the influential features in deriving the model’s output are shown. The features are listed on the y-axis in order of decreasing influence, while the x-axis reflects the model’s output value. A line depicts the prediction for each observation. Each line crosses the x-axis at the predicted value for the relevant observation at the top of the figure. The SHAP values of each parameter are added to the base value of the model from the bottom to the top of the plot, illustrating how each feature affects the final prediction. Features moving rightward positively influence the model, while those moving leftward have a negative effect. (D) Bar plot: displays the average absolute SHAP values for each characteristic, with the x-axis indicating that L3 SMA has the greatest contribution of all the features. (E) Waterfall plot: illustrates the degree to which each feature in a single sample has an effect on the model. The x-axis indicates the SHAP value, with the expected value of the model output shown at the bottom. Each row illustrates how the contribution of each feature shifts the value from the expected model output to the prediction. This graph shows that the TR (intervention type) feature has the highest positive influence on model prediction performance, while the L3 SMA feature has the largest negative effect. CG: control group; GS: grip strength; RSMI: relative skeletal muscle mass index; SHAP: Shapley additive explanations; SMA: skeletal muscle area; SMFA: skeletal muscle intramuscular fat area; STG: strength training group; TCSG: tai chi exercise and strength training hybrid group; TR: intervention type.

The SHAP values of features for contribution are presented in Figure 6. We used SHAP values specifically derived from the LightGBM model, as it demonstrated the best performance among all models. The feature’s shape value indicates the degree to which the feature contributes to the overall model. The greatest contributions were shown in grip strength and L3 SMA across all variables. It is clear from Figure 6B that the characteristics may have an effect on one another. This implies that by evaluating all possible combinations of features, the optimal combination of features that would increase the performance of the model can be identified.

## Discussion

### Principal Findings

This study suggests a novel hybrid training strategy for older individuals with sarcopenia that combines SET and tai chi. In addition, AI was used to foresee if the intervention in older adults will reverse their sarcopenia. The results of this study showed that at the end of the 24-week intervention, participants in both the TCSG and STG improved their grip strength, RSMI, and L3 SMA, albeit to varying degrees. In terms of L3 SMA, the TCSG outperformed the others and exhibited the most substantial improvement. The strongest results in reversion of sarcopenia, however, were seen with the hybrid exercise program combining the TCSG and STG. As well as this, we were among the first to use XAI to predict sarcopenia in a Chinese sample of older people with an average accuracy rate of 88.4% (SD 2%).

We revealed that older people with sarcopenia in the STG and TCSG showed improvements in hand grip strength, RSMI, L3 SMA, and L3 SMD after 24 weeks of intervention. In comparison to both the STG and CG, the L3 SMA of the patients in the TCSG significantly improved. In accordance with the present results, previous studies have demonstrated that SET intervention has the ability to reverse sarcopenia and improve physical health [66]. This result may be explained by the fact that the contraction of strength training’s mechanical stress has a variety of positive consequences that increase intracellular protein accumulation net-positively, leading to the remodeling of the extracellular matrix and the growth of muscle fibers [67]. Specifically, SET stimulates PGC-1α expression in a working muscle, which could induce IGF-1 and suppress myostatin, leading to significant skeletal muscle growth [68], and IGF-1 expression increased, which might prevent atrophy and encourage hypertrophy; this might be a contributing factor to the amelioration of skeletal muscle area. Meanwhile, mechanical loading encourages the expression of growth hormone, as the growth hormone/IGF-1 axis and enhanced IGF-1 gene expression are thought to be the primary mechanisms by which growth hormone regulates postnatal bone development.

Perhaps the most important finding is that in terms of L3 SMA and RSMI, the TCSG considerably outperformed the STG after 24 weeks. These findings corroborate those of prior research suggesting that hybrid exercise may have a more potent effect on muscles [39,69-71]. Chinese traditional exercise, an assortment of low-intensity cardio mind-body exercises such as tai chi, Yijin Jing, Baduanjin, etc, has been used in China for centuries to both prevent and treat illness. As an aerobic training with low intensity, tai chi is commonly regarded as that it can improve physical condition, including improving endurance ability, increasing insulin sensitivity, and modulating fat metabolism. This impact is principally linked to the skeletal muscle’s markedly increased mitochondrial volume and density. PGC-1α/FNDC5/UCP1 signaling pathway activation and PGC-1α overexpression are the 2 factors responsible for this increase [72]. This effect increases the amount and activity of mitochondrial enzymes while also increasing the rate of fat burning in muscles both at rest and during low-intensity exercise [73-76]. Another possible explanation for this is that the theoretical principles of traditional Chinese medicine are helpful in maintaining a calm and composed state of mind. They also help the body’s innate self-regulatory or self-repair mechanisms to trigger and help the body to release endogenous neurohormones in a balanced manner [77,78]. Thus, the hybrid exercise training protocol of SET and tai chi had better performance and greater advantages than other intervention strategies.

Further, 1 unexpected finding was the extent to which neither the hybrid program nor SET could prevent intramuscular fat buildup. It is commonly known that as people age, their intramuscular fat content gradually increases [79,80]. The L3 level of the skeletal muscle intramuscular fat area showed a growing tendency in all 3 groups following the exercise intervention, but no statistical significance was identified. The findings of this study do not support the previous research finding that strength training and aerobic activity might assist older persons in avoiding age-related intramuscular fat accumulation [81]. This finding may be attributable to differences in the intensity and frequency of aerobic activity between studies.

The discovery that a total of 29% (n=27) of the participants were able to reverse their sarcopenia is perhaps the most clinically important finding. This included 40% (n=12) of the STG and 45.5% (n=15) of the TCSG. Our study links recovery from geriatric disease with a mixed exercise intervention, and the findings of this study could provide a new option for the treatment of sarcopenia and broadly support other research efforts in similar areas linking geriatric disease recovery to hybrid exercise [69,71].

Several limitations need to be noted regarding this study. First, given that there has been a wealth of research performed on the benefits of tai chi on skeletal muscle, we did not set up a distinct tai chi intervention group. Second, despite its effectiveness in enhancing the skeletal muscle area and RSMI in geriatric individuals with sarcopenia, the hybrid exercise program has limitations in terms of skeletal muscle intramuscular fat area. Third, we believe individual factors, such as the severity of sarcopenia and the activity habits of older people with sarcopenia, may influence the reversibility of sarcopenia. This study’s sample size offers valuable insights, yet further validation in larger cohorts is recommended in alignment with the sample size guidelines proposed [82] for clinical prediction models prior to clinical implementation. By using initial sarcopenia in older individuals, reversal of sarcopenia, and interventions as foundational data, a more comprehensive system with diverse features may be developed in the future to provide specialized medical assistance to this patient population.

### Conclusions

According to the results of our research, a combination exercise program consisting of SET and tai chi is capable of increasing muscle mass and reversing sarcopenia in older people effectively. In addition, the LightGBM classifier model performed better in determining if sarcopenia in older people can be reversed.

## Supplementary material

10.2196/58175 Checklist 1 CONSORT-EHEALTH checklist (V 1.6.1).

## Abbreviations

AI: artificial intelligence
ASM: appendicular skeletal muscle mass
AUC: area under the curve
AWGS: Asian Working Group for Sarcopenia
CG: control group
CT: computed tomography
HU: Hounsfield units
IGF-1: insulinlike growth factor 1
L3: third lumbar
L3 SMA: skeletal muscle area at the third lumbar vertebra
L3 SMD: skeletal muscle density at the third lumbar vertebra
L3 SMFA: skeletal muscle intramuscular fat area at the third lumbar vertebra
L3 SMFD: skeletal muscle intramuscular fat density at the third lumbar vertebra
LR: logistic regression
MFI: muscle fat infiltration
QCT: quantitative computed tomography
RF: random forest
RM: repetition maximum
RSMI: relative skeletal muscle mass index
SET: strength exercise training
SHAP: Shapley additive explanations
STG: strength training group
TCSG: tai chi exercise and strength training hybrid group
XAI: explainable artificial intelligence
